# Validation and Psychometric Properties of the Minnesota Living With Heart Failure Questionnaire in Individuals With Coronary Artery Disease in Lithuania

**DOI:** 10.3389/fpsyg.2021.771095

**Published:** 2022-02-04

**Authors:** Julija Gecaite-Stonciene, Julius Burkauskas, Adomas Bunevicius, Vesta Steibliene, Jurate Macijauskiene, Julija Brozaitiene, Narseta Mickuviene, Nijole Kazukauskiene

**Affiliations:** ^1^Laboratory of Behavioral Medicine, Neuroscience Institute, Lithuanian University of Health Sciences, Palanga, Lithuania; ^2^Faculty of Nursing, Lithuanian University of Health Sciences, Kaunas, Lithuania

**Keywords:** Minnesota living with heart failure questionnaire, quality of life, factorial structure, measures, psychometrics, validation, coronary artery disease, cross-cultural

## Abstract

**Background:**

Health-related quality of life (HRQoL) is known to be impaired in individuals with coronary artery disease (CAD), especially in those after a recent acute coronary syndrome (ACS). Heart failure (HF) is a common burden in this population that significantly contributes to worsening HRQoL. To accurately measure the level of HRQoL in individuals with CAD after ACS, disease-specific scales, such as the Minnesota living with heart failure questionnaire (MLHFQ), are recommended. Nevertheless, to date, there has not been a study that would comprehensively evaluate the psychometric properties of the MLHFQ in a large sample of individuals with CAD after ACS. The debate regarding the internal structure of MLHFQ is also still present. Hence, this study aimed to translate the MLHFQ and evaluate its internal structure, reliability/precision, and validity in individuals with CAD following ACS in Lithuania.

**Methods:**

In the cross-sectional study, 1,083 participants (70% men, age *M* = 58, *SD* = 9) were evaluated for sociodemographic and clinical characteristics. HRQoL was measured using the MLHFQ and the Short Form-36 health survey (SF-36). In addition, exercise capacity (EC) was also evaluated in the study patients, using a standardized computer-driven bicycle ergometer.

**Results:**

The internal consistency of the MLHFQ subscales (0.79−0.88) was found to be good. Confirmatory factor analysis (CFA) provided the support for the three-factor model (“physical domain,” “social domain,” and “emotional domain”) of the MLHFQ and showed acceptable fit [comparative fit indices (CFI) = 0.894; goodness-of-fit (GFI) = 0.898; non-normal fit index (NFI) = 0.879, and root mean square error of approximation (RMSEA) = 0.073]. Regarding convergent evidence, significant associations were found between the MLHFQ domains and the SF-36 domains and EC (r’s range 0.11−0.58).

**Conclusion:**

The current study completed cultural validation and provided further information on the psychometric characteristics of the MLHFQ in Lithuania, suggesting MLHFQ as a valid and reliable instrument to measure HRQoL. The Lithuanian version of MLHFQ is best described by a three-factor solution, measuring physical, social, and emotional dimensions of HRQoL among individuals with CAD following ACS.

## Introduction

Coronary artery disease (CAD) is considered the leading cause of morbidity and mortality worldwide and is a major factor in the development and progression of heart failure (HF) (Mozaffarian et al., 2016). In the developed countries, approximately 1–2% of adults meet the criteria for HF; however, in people who are 70 years of age or older, the prevalence is ≥10% (Mosterd and Hoes, 2007). Given the aging population in Europe, the prevalence of HF is likely to rise (Iacoviello and Antoncecchi, 2013). Acute coronary syndrome (ACS), such as myocardial infarction (MI) and unstable angina pectoris (Amsterdam et al., 2014), is one of the most common presentations of CAD, leading to the increase of long-term incidence of HF up to 30% (Qureshi et al., 2018; Cordero et al., 2021). Moreover, one out of ten individuals after ACS that is considered in the category of a low-risk for development of HF, eventually develops a new-onset of HF (Cordero et al., 2021). Thus, even though they are distinct conditions, CAD, ACS, and HF often coexist together.

Health-related quality of life (HRQoL) in individuals with heart-related conditions is known to be impaired (Callus et al., 2020; Journiac et al., 2020). HRQoL is especially problematic in those with CAD after ACS with HF (Staniute et al., 2015; Kazukauskiene et al., 2019). According to the WHO, QoL is described as the perception of individuals’ position in life in the context of the culture and value systems in which they live and in relation to their objectives, expectations, concerns, and standards. In clinical settings, HRQoL has been described as the person’s perception of the influence that the illness has on their life. Specifically, HRQoL measures the person’s perception about living with the disease through functional capacity, occupational health, the general perception of health status, and psychological/social functioning in the context in which they operate (Haas, 1999; Erceg et al., 2013; Falk et al., 2013; Mogle et al., 2017).

To evaluate the HRQoL of an individual, generic and disease-specific instruments are commonly used. One of the most known disease-specific instruments for measuring HRQoL is the Minnesota Living with HF Questionnaire (MLHFQ) (Rector and Cohn, 1992; Garin et al., 2014). The MLHFQ has advantages over generic scales, as it has the responsiveness and the capacity to discriminate between different magnitudes of change in individuals’ HRQoL (Garin et al., 2009; Rajati et al., 2016; Napier et al., 2018; Gonzalez-SaenzdeTejada et al., 2019). This scale has been adapted and translated to at least 34 languages in various countries and has shown good psychometric properties (Sneed et al., 2001; Bennett et al., 2003; Heo et al., 2005; Ho et al., 2007; Garin et al., 2008, 2013, 2014; Moon et al., 2012; Bilbao et al., 2016; Zahwe et al., 2020). Yet, up to date, there is a lack of studies that would examine the reliability/precision and validity of the MLHFQ in a large sample of individuals with CAD following ACS. To our knowledge, there are no psychometric studies on the MLHFQ in the Lithuanian population as well.

Furthermore, with regard to the psychometric properties of the MLHFQ, studies that investigated its internal structure yielded inconsistent results. Specifically, some of the studies indicated a two-factor solution (i.e., physical and emotional dimensions) (Middel et al., 2001; Garin et al., 2013; Brokalaki et al., 2015; Bilbao et al., 2016) that includes the original MLHFQ study (Rector and Cohn, 1992). However, ample published studies that validated the three-factor internal structure of the MLHFQ also exist (Heo et al., 2005; Ho et al., 2007; Moon et al., 2012; Lambrinou et al., 2013; Munyombwe et al., 2014; Mogle et al., 2017; Barnett et al., 2019; Zahwe et al., 2020) that report the potential existence of a third factor of “social domain.” Even though the inconsistent findings might be attributed to a variety of methodological aspects and cultural differences, further studies in a large sample of individuals with HF are warranted.

Considering the knowledge gap in terms of psychometric properties of the MLHFQ, this study aimed to translate and evaluate the applicability, internal consistency, and validity of the MLHFQ and to investigate the internal structure when administered on individuals with CAD following ACS in Lithuania.

## Materials and Methods

### Study Procedure

In sum, 1,190 consecutive patients with CAD were invited to participate in the larger study during the period from February 2014 to January 2019. The inclusion criteria were (1) current diagnosis of ACS, as defined by acute MI or unstable angina pectoris, and (2) the participation in the cardiac rehabilitation program at Lithuanian University of Health Sciences, Neuroscience Institute, Hospital Palangos Klinika within 1–2 weeks following treatment for ACS.

The exclusion criteria were (1) unstable cardiovascular condition (*n* = 52), (2) other severe illness (e.g., kidney failure or musculoskeletal pathology) (*n* = 28), and (3) unwillingness to participate in the study (*n* = 27). In sum, the final sample was comprised of 1,083 subjects (76% men, age *M* = 57, *SD* = 9). All participants underwent standardized diagnostic and treatment procedures for secondary CAD prevention, based on the established guidelines (Gibbons et al., 2002; Piepoli et al., 2010; Fletcher et al., 2013; O’Gara et al., 2013).

Within 2 days of admission to the cardiac rehabilitation, all study participants were assessed for demographic (i.e., age, gender, education, and marital status) and clinical factors [i.e., New York Heart Association (NYHA) functional class, angina pectoris class, medical diagnosis, and body mass index (BMI)]. Standard echocardiography testing was performed to evaluate left ventricular ejection fraction (LVEF).

All study participants independently completed the self-report questionnaires. In terms of HRQoL scales, we used the MLHFQ (Rector and Cohn, 1992) and the 36-Item Short Form Medical Outcome Questionnaire (SF-36) (Ware and Sherbourne, 1992).

All procedures conducted in current research involving human subjects were in compliance with the ethical principles of the Biomedical Research Ethics Committee for Biomedical Research at Lithuanian University of Health Sciences and conformed to the principles outlined in the Declaration of Helsinki. Informed consent was attained from all individual participants included in the study.

### Measures

#### The Minnesota Living With Heart Failure Questionnaire

The MLHFQ is comprised of 21 items evaluating physical, social, and emotional aspects of life, referring to weaknesses often associated with the cardiac insufficiency profile (Rector and Cohn, 1992). The major advantage of the MLHFQ for those with HF, in comparison to other generic HRQoL assessment scales, is that it specifically addresses the daily living challenges and context of those experiencing HF.

The questions are based on a six-point Likert scale from 0 (“no impact of HF on HRQoL”) to 5 (the significant negative impact of HF on HRQoL). The MLHFQ has a total score (21 items, score range 0–105), the sum of points of the physical dimension subscale (8 items, score range 0–40), and the sum of points of the emotional dimension subscale (5 items, scores range 0–25). Higher scores indicate worse HRQoL.

Permission to translate the original MLHFQ into a Lithuanian version was obtained from the University of Minnesota, which holds its copyright. The questionnaire was translated by the two independent bilingual translators. The third bilingual translator without any previous knowledge about the MLHFQ back-translated and re-conciliated the Lithuanian version. The final version was completed after the revisions were provided by several different experts in the field that were fluent in both languages, i.e., the cardiologist (Julija Brozaitiene), the medical psychologists (Julija Gecaite-Stonciense and Julius Burkauskas), and the registered nurse (Nijole Kazukauskiene).

#### 36-Item Short Form Medical Outcome Questionnaire

The SF-36 is comprised of 8 multi-item scales that evaluate HRQoL on 8 domains: (1) physical functioning, (2) social functioning, role limitations due to (3) emotional problems and (4) physical problems, (5) mental health, (6) energy/vitality, (7) pain, and (8) general health perception. Each SF-36 domain is scored from 0 to 100, where higher scores reflect better HRQoL (Ware and Sherbourne, 1992). Internal-consistency reliability (Cronbach’s α) of eight subscales has been found to range between 0.57 and 0.85.

#### Exercise Capacity Testing

In addition, study participants also completed testing for EC. Participants’ (EC) was measured by the cardiologist (Julija Brozaitiene) using a standardized computer-driven bicycle ergometer with workload rising by 25 watts (W) every 3 min (Fletcher et al., 2013). The peak of workload (PW) in watt (W) or metabolic equivalent of task (MET) (1 MET = 3.5 ml of oxygen uptake per kilogram of body weight per min) at the completion of the test reflected EC. Detailed procedures of EC have been reported in our study elsewhere (Kazukauskiene and Burkauskas, 2018). Reduced EC was regarded as ≤50 W, as this cut score is associated with moderate-to-severe functional impairment and cardiac symptoms (Gibbons et al., 2002; Piepoli et al., 2010; Fletcher et al., 2013).

### Statistical Analysis

Statistical analysis was performed using the Statistical Package for Social Sciences, SPSS Statistics for Windows, Version 22.0.0.0 (IBM SPSS Statistics for Windows, Version 22.0. Armonk, NY, United States: IBM Corp). Data are expressed as a mean ± SD for continuous variables and as a number (percent) for qualitative variables. The distribution of measures was assessed using skewness and kurtosis analysis. In a total sample, the scores were approximately normally distributed for the total score and the physical subscale with skewness of 0.518 (SE = 0.325) and kurtosis of − 0.280 (SE = 0.149) for the total score and skewness of 0.565 (SE = 0.341) and kurtosis − 0.496 (SE = 0.264) for the physical subscale. Values of the emotional subscale were 1.133 (SE = 0.074) for skewness and 0.893 (SE = 0.27) for kurtosis, respectively. Taking into account the large sample size of this current study, the latter scale was considered to be normally distributed (Kim, 2013).

Cronbach’s α coefficients were calculated to evaluate the internal-consistency reliability of the MLHFQ and its dimensions and to contrast these with the SF-36 scales and composites. For each of the subscales of the MLHFQ and SF-36 domains, the floor and ceiling effects were described as the proportion of individuals at each subscale, who achieved the lowest or the highest possible score. The floor and ceiling effects were considered to be present when at least 15% of respondents received the lowest or the highest rating, respectively.

Convergent evidence was evaluated by measuring the Pearson Correlation Coefficients of the MLHFQ and its dimensions and all the scores of the SF-36 subscales. It was expected that the MLHFQ total score would be highly associated with the SF-36 vitality and social functioning, while scores of the MLHFQ dimension pertaining to physical health would highly correlate with the SF-36 physical functioning, vitality, and social functioning. Further, it was expected that the MLHFQ dimension pertaining to emotional health would be highly correlated with the SF-36 emotional wellbeing and vitality, respectively.

Construct validity of the MLHFQ was evaluated by comparing the MLHFQ and its scores on a different dimension to age (<65 years vs. ≥65 years), gender (male, female), NYHA functional class (NYHA I–II class vs. NYHA III–IV class), LVEF (LVEF ≤ 40% vs. LVEF >40%), EC (≤50 W vs. >50 W), and BMI (<30 kg/m^2^ vs. ≥30 kg/m^2^) using the Independent Samples *t*-test.

Exploratory factor analyses (EFA) (sub−sample 1, *n* = 541), sample adequacy, and factorability of data were analyzed using the Kaiser-Meyer-Olkin (KMO) measure and the Bartlett test for sphericity. We performed an initial EFA to determine the dimensionality of the MLHFQ questionnaire using principal component analysis with the varimax rotation method. Cut scores for factor loadings were set at 0.4. Items cross-loading on multiple factors were assigned to the factor with higher loading. We used confirmatory factor analysis (CFA) (sub−sample 2, *n* = 542) to determine the internal structure of the MLHFQ using maximum likelihood (ML) estimation. The fitness of the model with the data was measured by calculating the absolute and comparative fit indices (CFI). Absolute fit indices included chi-square goodness-of-fit (GFI), non-normal fit index (NFI), and root mean square error of approximation (RMSEA).

## Results

In terms of descriptive information, Table 1 represents the basic sociodemographic characteristics of all individuals participating in the study. In summary, the majority of study participants (79%) had moderate HF symptoms (NYHA II functional class), 14% had severe HF symptoms, and were assigned to NYHA III–IV functional class. The minority of the participants (7%) had mild HF symptoms (NYHA I functional class). In sum, 28% of study participants had angina pectoris, 61% had acute MI, and 11% had previous MI. Eighty-nine percent of individuals were treated with beta-blockers, 94% – statins, 81% – angiotensin-converting enzyme inhibitors, and 15% – diuretics, and other medications.

**TABLE 1:** Characteristics of all study patients.

	Total group
	***n* = 1083**
Age, years (mean ± SD)	57.44 ± 9.03
**Gender, *n* (%)**	
Male	821 (75.8)
Female	262 (24.2)
**Marital status, *n* (%)**	
Cohabiting	904 (83.5)
Single	21 (1.9)
Divorced	79 (7.3)
Widowed	79 (7.3)
**Education, *n* (%)**	
Up to 8 years	85 (7.8)
High school graduate	536 (49.5)
College/university degree	462 (42.7)
Body mass index ≥ 30 kg/m^2^, *n* (%)	484 (44.7)
**Medical diagnosis, *n* (%)**	
Angina pectoris	302 (27.9)
Acute myocardial infarction	661 (61.0)
Previous myocardial infarction	120 (11.1)
**New York Heart Association functional class, *n* (%)**	
I	74 (6.8)
II	859 (79.3)
III-IV	150 (13.9)
**Left ventricular ejection fraction, *n* (%)**	
≤40%	121 (11.2)
>40%	962 (88.8)
**Exercise capacity, *n* (%)**	
≤50 W	437 (40.4)
>50 W	646 (59.6)
**Medications, *n* (%):**	
Nitrates	270 (24.9)
Angiotensin-converting-enzyme inhibitors	876 (80.9)
Beta-blockers	964 (89.0)
Diuretics	158 (14.6)
Statines	1015 (93.7)
Benzodiazepines	173 (16.0)

Mean scores of the MLHFQ and the SF-36 domains are shown in Table 2. The lowest SF-36 score was for the role limitations due to physical problems subscales, while the highest score was for the subscale of emotional wellbeing. Cronbach’s coefficients α were greater than 0.70 for all subscales, except the subscales of general health (0.688) and social functioning (0.572). In the current study, the MLHFQ had adequate internal-consistency reliability (Cronbach’s α of Total score = 0.91, Cronbach’s α of physical dimension = 0.88, and emotional dimension = 0.82).

**TABLE 2:** Health-related quality of life scales characteristics.

HRQoL measure	No. of items	Mean ± SD	Median [IQR]	Min	Max	Ceiling, *n* (%)	Floor, *n* (%)	Cronbach α
*MLHFQ*			*Score*		*Score*			
Total score	21	31.33 ± 19.84	29 (16–45)	0	100	26 (2.4)	0	0.912
Physical dimension	8	12.89 ± 9.28	12 (5–20)	0	40	62 (5.7)	2 (0.2)	0.885
Emotional dimension	5	5.72 ± 5.29	4 (2–9)	0	25	158 (14.6)	3 (0.3)	0.828
*SF-36*								
Physical functioning	10	67.75 ± 20.16	70 (55–85)	0	100	3 (0.3)	37 (3.4)	0.859
Role limitations due To physical problems	4	29.18 ± 36.87	0 (0–50)	0	100	557 (51.4)	153 (14.1)	0.829
Role limitations due to emotional problems	3	52.85 ± 43.74	66.67 (0–100)	0	100	363 (33.5)	437 (40.4)	0.851
Social functioning	2	66.66 ± 26.47	66.67 (44.44–88.89)	0	100	7 (0.6)	231 (21.3)	0.572
Emotional well-being	5	68.13 ± 19.33	72 (56–84)	4	100	0	45 (4.2)	0.800
Vitality	4	58.36 ± 21.02	60 (45–75)	0	100	2 (0.2)	20 (1.8)	0.718
Pain	2	50.35 ± 27.62	44.44 (33.33–66.67)	0	100	62 (5.7)	103 (9.5)	0.752
General health	5	52.89 ± 18.69	50 (40–65)	0	100	5 (0.5)	6 (0.6)	0.688

*HRQoLf, health-related quality of life; IQR, interquartile range; min, lowest; max, highest; MLHFQ, Minnesota Living with Heart Failure Questionnaire; SF-36, Medical Outcomes Study 36-Item Short Form Health Survey; α, Cronbach’s alpha coefficient.*

Table 2 also shows the ceiling (“poorest” HRQoL) and floor (“best” HRQoL) effects of the MLHFQ. The ceiling effect was present for the total score of the MLHFQ 2.4% (*n* = 26), physical dimension 5.7% (*n* = 62), and emotional dimension 14.6% (*n* = 158). The floor effect was present for the physical dimension 0.2% (*n* = 2) and emotional dimension of the MLHFQ 3% (*n* = 3). In contrast, the SF-36 ceiling represents “best” HRQoL, while floor indicates “poorest” HRQoL effects. The ceiling effect was present for the SF-36 subscales of physical functioning 3% (*n* = 3), role limitation due to physical problems 51.4% (*n* = 557), role limitations due to emotional problems 33.5% (*n* = 363), social functioning 6%, (*n* = 7), vitality 2% (*n* = 2), pain 5.7%, (*n* = 62), and general health 5% (*n* = 5). Floor effect was present for the SF-36 subscales of physical functioning 3.4% (*n* = 37), role limitation due to physical problems 14.1% (*n* = 153), role limitations due to emotional problems 40.4% (*n* = 437), social functioning 21.3%, (*n* = 231), emotional wellbeing 4.2% (*n* = 45), vitality 1.8% (*n* = 20), pain 9.5%, (*n* = 103), and general health 6% (*n* = 6).

As demonstrated in Table 3, the MLHFQ total score was mostly associated with the SF-36 vitality (*r* = − 0.597, *p* < 0.001) and the SF-36 social functioning (*r* = − 0.594, *p* < 0.001), while scores on the MLHFQ dimension pertaining to physical health were mostly associated with the SF-36 physical functioning (*r* = − 0.571, *p* < 0.001), SF-36 social functioning (*r* = − 0.577, *p* < 0.001), and SF-36 vitality (*r* = − 0.593, *p* < 0.001). The MLHFQ dimension pertaining to emotional health was mostly associated with the SF-36 emotional wellbeing (*r* = − 0.646, *p* < 0.001) and the SF-36 vitality (*r* = − 0.576, *p* < 0.001), respectively.

**TABLE 3:** Convergent evidence of the Minnesota Living with Heart Failure Questionnaire (MLHFQ), 36-Item Short Form Health Survey (SF-36), and exercise capacity in the overall sample.

	MLHFQ		
	Total score	Physical dimension	Emotional dimension
**SF-36**			
Physical functioning	−0.520 (<0.001)	−0.571 (<0.001)	−0.402 (<0.001)
Role limitations due to physical problems	−0.402 (<0.001)	−0.387 (<0.001)	−0.267 (<0.001)
Role limitations due to emotional problems	−0.375 (<0.001)	−0.323 (<0.001)	−0.372 (<0.001)
Social functioning	−0.594 (<0.001)	−0.577 (<0.001)	−0.451 (<0.001)
Emotional well-being	−0.524 (<0.001)	−0.445 (<0.001)	−0.646 (<0.001)
Vitality	−0.597 (<0.001)	−0.593 (<0.001)	−0.576 (<0.001)
Pain	−0.454 (<0.001)	−0.460 (<0.001)	−0.319 (<0.001)
General health	−0.516 (<0.001)	−0.469 (<0.001)	−0.476 (<0.001)
Exercise capacity	−0.248 (0.000)	−0.289 (0.000)	−0.208 (0.000)

*SF-36, Medical Outcomes Study 36-Item Short Form Health Survey.*

The strong associations were observed between theoretically similar domains, for example, the MLHFQ physical dimension and the SF-36 measures of physical HRQoL, and between the MLHFQ emotional dimension and the SF-36 measures of mental HRQoL, providing the evidence for convergent evidence (Table 3).

Table 4 shows the EFA of the MLHFQ items. To determine the internal structure, all 21 items of the MLHFQ underwent an EFA with data of sub−sample 1 (*n* = 541). A value of 0.926 on the KMO test indicated adequate correlation matrices. The Bartlett sphericity test was significant at χ^2^ = 4882,855 (*p* < 0.001), indicating the presence of significant correlations and reinforcing the relevance of the factor analysis. In all three factors, the multiple loadings of items had factor-loading values of >0.40. Factor analysis with three-factor explained 52.6% of the total variance, out of which 36.7% was explained by the first factor, 8.8% by the second factor, and 7.1% by the third factor. Items 1–6, 12, and 13 loaded heavily onto the first factor. Items 16–21 loaded on to the second factor. Items 7–11 and 14 loaded heavily onto the third factor. Item 15 did not load on any of the subscales or the overall factor and was eliminated from the CFA analysis. CFA using data of sub−sample 2 (*n* = 542) confirmed the EFA solution. CFA showed acceptable fit [CFI = 0.895; GFI = 0.882; NFI = 0.864, RMSEA = 0.074 95% CI = (0.068;0.080)].

**TABLE 4:** Factor analysis of the MLHFQ items.

Item		Factor 1 (physical)	Factor 2 (emotional)	Factor 3 (social)
1	Swelling in your ankles, legs	0.434	0.183	−0.084
2	Resting during day	0.720	0.140	0.286
3	Walking or climbing stairs difficult	0.765	0.115	0.297
4	Working around house difficult	0.602	0.111	0.548
5	Away from home difficult	0.647	0.100	0.444
6	Sleeping difficult	0.539	0.312	0.157
7	Relating to or doing things with friends	0.419	0.218	0.545
8	Working to earn a living difficult	0.199	0.062	0.670
9	Recreational activities difficult	0.373	0.088	0.726
10	Sexual activities difficult	0.003	0.064	0.734
11	Eating less foods, I like	0.126	0.258	0.508
12	Shortness of breath	0.651	0.212	−0.009
13	Fatigue	0.724	0.366	0.180
14	Hospitalization	0.001	0.304	0.516
15*	Medical costs	0.396	0.378	0.246
16	Side effects from medications	0.193	0.529	0.173
17	Feeling burden to family or friends	0.011	0.479	0.401
18	Feeling loss of self-control	0.160	0.773	0.126
19	Being worried	0.239	0.753	0.142
20	Difficulty concentrating or remembering	0.418	0.682	0.043
21	Being depressed	0.268	0.767	0.157
Cronbach’s α		0.864	0.863	0.827

*Data refer to subsample 1 of sample 2.*

*Item not belonging to any factor.

*MLHFQ, Minnesota Living with Heart Failure Questionnaire.*

Table 5 provides Pearson Correlation Coefficients between the factors of the MLHFQ, the SF-36, and EC. As expected, the scores of the factors were significantly correlated with the SF-36 and EC, respectively, supporting convergent evidence.

**TABLE 5:** Convergent evidence: Pearson correlation coefficients between the factors of Minnesota Living with Heart Failure Questionnaire (MLHFQ), 36-Item Short Form Health Survey (SF-36), and exercise capacity in the overall sample.

	Factors		
	1	2	3
**SF-36**			
Physical functioning	−0.575 (<0.001)	−0.331 (<0.001)	−0.406 (<0.001)
Role limitation due to physical problems	−0.379 (<0.001)	−0.355 (<0.001)	−0.271 (<0.001)
Role limitation due to emotional problem	−0.324 (<0.001)	−0.284 (<0.001)	−0.367 (<0.001)
Social functioning	−0.544 (<0.001)	−0.514 (<0.001)	−0.460 (<0.001)
Mental health	−0.442 (<0.001)	−0.323 (<0.001)	−0.622 (<0.001)
Energy/vitality	−0.594 (<0.001)	−0.370 (<0.001)	−0.571 (<0.001)
Bodily pain	−0.458 (<0.001)	−0.357 (<0.001)	−0.320 (<0.001)
General health	−0.475 (<0.001)	−0.355 (<0.001)	−0.486 (<0.001)
Exercise capacity	−0.294 (<0.001)	−0.112 (<0.001)	−0.215 (<0.001)

*MLHFQ, Minnesota Living with Heart Failure Questionnaire; SF-36, medical outcomes study 36-item short form health survey;*

*Factor 1, physical dimension.*

*Factor 2, social dimension;*

*Factor 3, emotional dimension.*

**FIGURE 1:**
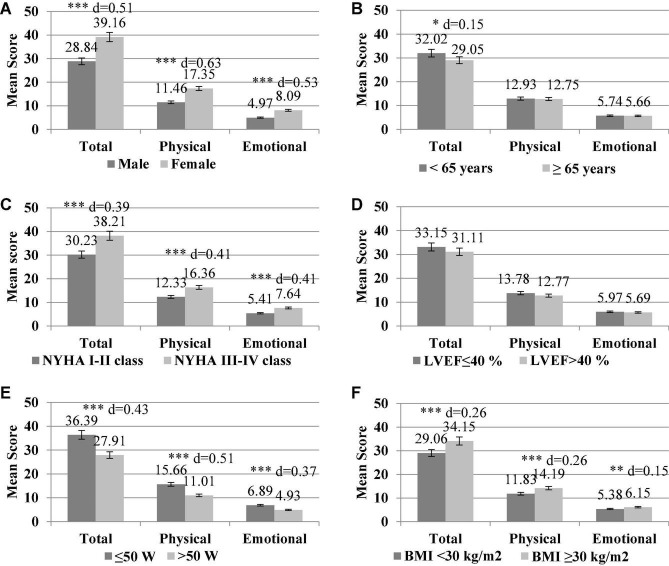
The differences of mean scores of the Minnesota living with heart failure questionnaire (MLHFQ) dimensions (global, physical, and emotional health) according to gender **(A)**, age **(B)**, New York Heart Association, NYHA functional class **(C)**, left ventricular ejection fraction, LVEF **(D)**, watts for exercise capacity **(E)**, and body mass index and BMI **(F)**. Effect size **(D)** for the difference between the groups. (^∗^*p* < 0.05; ^∗∗^*p* < 0.01; ^***^p < 0.001; d, Cohen’s d effect size).

In younger women, the higher NYHA functional class and reduced EC were associated with the higher MLHFQ scores, indicating worse HRQoL. Individuals younger than 65 years scored significantly higher only on the MLHFQ total score (*p* < 0.05, with a moderate effect size). Female gender, higher NYHA functional class, obesity (BMI ≥ 30 kg/m^2^), and reduced EC were linked with higher scores on all dimensions of the MLHFQ (*p* < 0.001, with a small-to-moderate effect size), indicating individuals’ worse HRQoL. The comparison analyses showed no differences between LVEF ≤ 40% vs. LVEF ≤40% groups (Figure 1).

## Discussion

Our results indicated that the MLHFQ could be considered as a valid and reliable instrument for the evaluation of HRQoL in individuals with CAD following ACS during a cardiac rehabilitation program in Lithuania. The current data support sound psychometric properties of MLHFQ and its response to therapeutic interventions. The current findings also suggest the existence of the third factor in the MLHFQ in individuals with CAD following ACS, some of whom lack HF symptoms.

To the best of our knowledge, this is the first study assessing the performance of the MLHFQ among individuals with CAD undergoing ACS and is among the very few (Garin et al., 2008, 2013) which have included a comparison with the SF-36.

The MLHFQ’s three-factor internal-consistency reliability was good, which is in line with the performance of this instrument in randomized clinical trials performed in individuals with HF (Heo et al., 2005; Ho et al., 2007). For the distinct physical and emotional factors described in the original MLHFQ validation, empirical support was found (Rector and Cohn, 1992).

In addition, it is important to note that in our study the MLHFQ had relatively mild ceiling and floor effects, which are important psychometric properties of any scale (Feeny et al., 2013). Based on the current results, the MLHFQ can be considered as an applicable instrument to measure relatively minor changes in the self-reported health status of individuals with CAD after ACS representing symptoms of HF. The required threshold rate for floor and ceiling effects was not reached for the MLHFQ and their dimensions suggesting a good sensitivity of the MLHFQ for evaluating perceived HRQoL of individuals with CAD who underwent ACS.

Further, the strong correlations between theoretically related dimensions of the MLHFQ and the SF-36 have provided evidence of convergent evidence; results that also give the support for the underlying constructs supporting the instrument’s criterion validity in this population (Boateng et al., 2018). The MLHFQ physical and emotional dimensions were strongly associated with similar domains of the SF-36 measures of physical and mental HRQoL (Garin et al., 2008, 2013). In terms of objective measures, the MLHFQ was associated with EC, especially the physical dimension of MLHFQ and EC, indicating that EC plays a major role in decreasing HRQoL in those individuals (Bussoni et al., 2013).

Previous studies have suggested that the functional classification of NYHA was characterized by the severity of the symptoms of HF. Thus, it was expected that the MLHFQ would be useful for individuals with different HF severity levels (Carvalho et al., 2009). In the current study, the NYHA classification and the value of EC measured as external work in watts were consistent with higher MLHFQ scores, indicating worse HRQoL. A significant correlation between the NYHA functional classification and the score for the Spanish version of the MLHFQ was recorded in an earlier review (Parajon et al., 2004). Parajon et al. (2004) found that higher scores on the MLHFQ, suggesting lower HRQoL, were correlated with higher NYHA functional class and hospital admissions over the previous year. In a small sample of individuals with advanced chronic HF, Morcillo et al. (2007) reported strong associations between the MLHFQ, a functional class, and the SF-36. The findings were similar in our study as well.

Further, our study suggests the presence of a third “social” dimension of the MLHFQ. Among the other three-factor models, the best results obtained with this model were proposed by Garin et al. (2013). With regard to the internal structure, our study is also in line with other previous reports where HF was mostly a primary condition (Heo et al., 2005; Ho et al., 2007; Moon et al., 2012; Lambrinou et al., 2013; Munyombwe et al., 2014; Mogle et al., 2017; Barnett et al., 2019; Zahwe et al., 2020). Some of those studies (Heo et al., 2007; Mogle et al., 2017; Zahwe et al., 2020) had issues that fully support the psychometric soundness of several items. In our case, similar to Mogle et al. (2017) and Zahwe et al. (2020) reports, item 15 (“medical costs”) was problematic and did not fall under any of the factors. We assume that this issue can be partially attributed to cultural socioeconomic differences across countries, such as variations in healthcare policies across nations, which, depending on whether the country has a universal healthcare system, makes this item more or less important.

In addition, in the current study, sample disequilibrium was observed, as the majority (75.8%) of the participants were men with a mean age of 57.4. It is well established that CAD is more prevalent among men than women (Khan et al., 2020) and usually tends to develop 7–10 years earlier (Maas and Appelman, 2010; Piepoli, 2017). These reasons may partly explain the gender misbalance that was present in our study. In terms of age, a recent meta-analysis (Dibben et al., 2018) has reported the results of 47 studies performed in cardiac rehabilitations, suggesting the average age (58.3) in the study patients is close to the average age found in our study. Thus, the sample disequilibrium might be attributed to the reality of cardiac rehabilitations and their patients’ sociodemographic profiles across different countries.

The present study has several strengths, such as the large sample size and the use of validated instruments. The study has also measured EC by employing a standardized computer-driven bicycle ergometer that allowed to objectively assess the cardiac function of the study patients and compare it with the MLHFQ results. In terms of research limitations, the study was completed in a single rehabilitation clinic; thus, the results are found in a selective individuals’ group. Particularly, most of the study participants had mild-to-moderate HF, the significant majority were men, and all of them attended a single in-patient center, which may limit the generalization of our results. Thus, the findings should be interpreted with caution, when considering individuals with more advanced HF or individuals who do not attend rehabilitation programs after ACS.

Our study provided further knowledge of MLHFQ psychometric properties that may bolster the clinical knowledge of the application of this specific instrument in patients with CAD after ACS commonly presenting HF symptoms. This instrument may help to not only assess the subjectively perceived HRQoL but also provide the benefit of patient-centered care to improve individual health outcomes and deliver more holistically appropriate interventions and customized medical care plans during cardiac rehabilitation. Also, culturally specific evaluation instruments can improve the communication between clinicians and patients that may result in more accurate symptom assessment and effective treatment. According to our results, Lithuanian clinicians may reconsider item 15 (“medical costs”) during the assessment of HRQoL in cardiac rehabilitations, as it does not lead to any of the MLHFQ subscales and does not support the internal structure of this instrument.

## Conclusion

This research offers additional details about the validation and psychometric properties of the MLHFQ in Lithuania. The MLHFQ is a reliable and valid instrument for measuring HRQoL in individuals with CAD following ACS. Our study also supports a three-factor solution for the MLHFQ that includes “physical,” “social,” and “emotional” dimensions. The study provides support for the MLHFQ potential use for future research and clinical practice in individuals undergoing cardiac rehabilitation with HF.

## Data Availability Statement

The study dataset is available upon reasonable request to NK, nijole.kazukauskiene@lsmuni.lt. The completed license agreement to use the MLHFQ can be provided upon request to the corresponding author. Due to the copyright regulations, the Lithuanian version of the MLHFQ can be provided upon the request to Laboratory of Behavioral Medicine, Neuroscience Institute, Lithuanian University of Health Sciences after the license agreement with University of Minnesota is completed.

## Ethics Statement

The studies involving human participants were reviewed and approved by Biomedical Research Ethics Committee for Biomedical Research at Lithuanian University of Health Sciences. The patients/participants provided their written informed consent to participate in this study.

## Author Contributions

NK contributed to conceptualization, methodology, formal analysis, writing – review and editing, and investigation. JG-S contributed to writing – original draft, conceptualization, and investigation. JBu contributed to project administration, investigation, and writing – review and editing. JBr contributed to conceptualization and supervision. AB contributed to writing – review and editing. NK, JM, and VS contributed to supervision. All authors contributed to the article and approved the submitted version.

## Conflict of Interest

JG-S served as a consultant at FACITtrans. In the past several years JBu worked as a consultant to Cogstate, Ltd. The remaining authors declare that the research was conducted in the absence of any commercial or financial relationships that could be construed as a potential conflict of interest.

## Publisher’s Note

All claims expressed in this article are solely those of the authors and do not necessarily represent those of their affiliated organizations, or those of the publisher, the editors and the reviewers. Any product that may be evaluated in this article, or claim that may be made by its manufacturer, is not guaranteed or endorsed by the publisher.
